# Deep Reinforcement Learning for Edge Caching with Mobility Prediction in Vehicular Networks

**DOI:** 10.3390/s23031732

**Published:** 2023-02-03

**Authors:** Yoonjeong Choi, Yujin Lim

**Affiliations:** Department of IT Engineering, Sookmyung Women’s University, Seoul 04310, Republic of Korea

**Keywords:** vehicular network, edge caching, deep reinforcement learning, long short-term memory

## Abstract

As vehicles are connected to the Internet, various services can be provided to users. However, if the requests of vehicle users are concentrated on the remote server, the transmission delay increases, and there is a high possibility that the delay constraint cannot be satisfied. To solve this problem, caching can be performed at a closer proximity to the user which in turn would reduce the latency by distributing requests. The road side unit (RSU) and vehicle can serve as caching nodes by providing storage space closer to users through a mobile edge computing (MEC) server and an on-board unit (OBU), respectively. In this paper, we propose a caching strategy for both RSUs and vehicles with the goal of maximizing the caching node throughput. The vehicles move at a greater speed; thus, if positions of the vehicles are predictable in advance, this helps to determine the location and type of content that has to be cached. By using the temporal and spatial characteristics of vehicles, we adopted a long short-term memory (LSTM) to predict the locations of the vehicles. To respond to time-varying content popularity, a deep deterministic policy gradient (DDPG) was used to determine the size of each piece of content to be stored in the caching nodes. Experiments in various environments have proven that the proposed algorithm performs better when compared to other caching methods in terms of the throughput of caching nodes, delay constraint satisfaction, and update cost.

## 1. Introduction

Numerous things are connected to the Internet, which helps it to provide unlimited services to users. In particular, vehicles connected to the Internet are expected to grow faster by 2023 compared to other applications [1]. Connected vehicles have become possible due to multiple sensors of vehicles and the development of both intravehicle and intervehicle communication. Connected vehicles can communicate with everything and provide various services, such as road security notification and management, navigation systems, and media sharing [2]. In addition, infotainment such as streaming videos or audio that the users are interested in and information on the local environment are provided to vehicle users [3]. However, the download speed of video and audio contents, which are received from a remote server is decreased, and the backhaul burden is increased. If caching is performed at a distance closer to the vehicle user, this problem can be alleviated by distributing the centralized requests.

Cacheable spaces, which are located closer to the vehicle user, include road side units (RSUs) and vehicles. The installation of a mobile edge computing (MEC) server in the RSU creates space for the caching content, further enabling the delivery of the content from a location closer than that of the remote server. Caching to the RSUs decreases the latency and relieves the backhaul burden [4]. However, because the vehicle is constantly moving, the residence time in the RSU range is relatively shorter and the network topology continues to vary [5]. The vehicle provides a space with the on-board unit (OBU) to store the content. Caching in vehicles and receiving the content through device-to-device (D2D) communication reduces the burden of backhaul without installing additional infrastructure. However, compared to the RSU, the communicable distance of vehicles is shorter, and the storage space of vehicles is smaller [6]. If both RSUs and vehicles are used to cache content, their problems would be complementary to each other. However, both the MEC server of the RSU and OBU of the vehicle have limited caching capacity compared to the remote server; therefore, determining the content that has to be cached is an important issue.

There are several factors that affect the caching performance, one of which is the structure of the RSU network. Caching a variety of content when the coverage for RSUs is overlapped and caching popular content when it is not overlapped can increase the hit ratio [7]. In urban environments, infrastructures are densely installed, which create areas where the coverage of RSUs overlaps. To determine the cache content for vehicle users in an urban environment, it is necessary to consider the diversity of the content between the RSUs with overlapping ranges. The mobility of the vehicle also affects the content caching to the RSU. With respect to the movement of the vehicle, there are cases in which several vehicles gather at a specific RSU or stay for a shorter time period. When user demand is high, a more popular content is mainly requested, whereas when user demand is low, a less popular content is mainly requested [8]. Therefore, earlier identification of the area where vehicles are crowded would help to cache the appropriate content for each RSU.

In this study, we propose a caching strategy that uses both the RSU and vehicle as caching nodes. We aim to maximize the throughput from caching nodes because the distribution of content requests to caching nodes can reduce the content delivery time and backhaul burdens. To achieve the goal, the location of vehicles was predicted through LSTM and we tried to reduce duplicated content between overlapping RSUs. We adopted the deep deterministic policy gradient (DDPG) method, which is a deep reinforcement learning method, for two reasons. The first is to deal with a complex environment: for example, the location of the vehicle changes over time, and the caching nodes that could be connected to the vehicles vary depending on the location of the vehicle. Second, a continuous space can be provided by the DDPG. For each time slot, the content popularity and the number of vehicles in the service area of RSUs were defined as the states of a Markov Decision Process (MDP). The agent of the DDPG understood the environment and learned the appropriate caching strategy. We used a coded caching technique, and the coded caching is used to store the content in a divided form. Coded caching makes the action space continuous, because the content is stored in a segmented form.

The movement of the vehicle is considered through the process of predicting the mobility of the vehicle using long short-term memory (LSTM) to determine the number of vehicles in each RSU. The LSTM can memorize long-term and short-term memories. Because the data used for prediction consisted of the current and past trajectories of the vehicles, LSTM was selected as the prediction model. The main contributions of this study are as follows:

(1) We designed a content-caching scheme using RSUs and vehicles as caching nodes. There are both advantages and disadvantages associated with this. The vehicles and RSUs were configured to act as complementary each other.
(2) An LSTM-based vehicle location prediction model is designed to estimate the number of vehicles staying in the service area of each RSU. Based on the latitude and longitude coordinates of vehicles, future coordinates could be predicted through past trajectories of real vehicle mobility data. The number of vehicles in the RSU coverage at each time is computed by the LSTM model and this information is forwarded to the caching algorithm.
(3) A DDPG-based caching algorithm is proposed to effectively use caching nodes with limited capacity in the urban environment. The agent of DDPG decides the types and sizes of content to cache in RSUs and vehicles. The diversity of contents in areas where the service ranges of RSUs are overlapped, and the popularity of contents that changes over time is identified to ensure efficient caching strategy. To maximize the caching nodes throughput, an appropriate MDP is designed by considering the time-varying content popularity and duplicate content.

The remainder of this paper is organized as follows: Section 2 analyzes the related works. Section 3 describes the system model, and Section 4 proposes a DDPG-based caching algorithm. In Section 5, the comparison of the performance improvement of the delay constraint satisfaction, the update cost and the edge throughput with respect to other algorithms is depicted through experiments. Finally, Section 6 presents the conclusions and future research directions.

## 2. Related Works

Several studies have been conducted on caching algorithms for vehicle users who request contents or files. Facilities that can cache at a nearer proximity to vehicles than remote servers can include fixed spaces such as the RSU, small base station (SBS), and macro base station (MBS). In addition, vehicles can cache content and form ad-hoc networks.

In the case of RSU and SBS, because they are fixed in one place, the content could be cached by installing RSU and SBS at a location where the vehicle stays for a relatively longer period of time. In [9], RSUs were installed at an intersection where the residence time of vehicles was long. By considering the amount of time the vehicle stays in the RSU, the number of vehicles staying in the RSU, and bandwidth, Ref. [9] decides whether to store the file chunk to maximize the hit probability. The vehicles download file chunks as they pass through the routes and recover the original file after they obtain all chunks. In [10], another caching method was proposed, in which the files were divided according to the vehicle movement to reduce the duplicated content and backhaul burden. Each RSU was installed in the direction in which the vehicle traveled, and the content size to be cached was determined based on the probability of the direction in which the vehicle moved. In [11], proactive caching was used to solve problems caused by vehicle mobility. This was because the mobility of vehicles significantly decreased the residence time in one RSU, thereby making it difficult for the vehicles to download the content. In addition, federated learning was applied to determine popular contents while protecting the personal information of the vehicle users. In [12], a caching algorithm was proposed to minimize the RSU cache update cost and file download cost. Because the speeds of vehicles and RSU file are different, two time-scale models were used. Ref. [12] determined the content popularity over time. This method maintained the balance between changes in the popularity and updates of contents.

The advantage of ad hoc networks composed of vehicles is that they serve as caching nodes while moving. This is because the requestor can stay closer to the caching node for a longer period of time if the requesting vehicle has a similar route. In [13], a cache replacement algorithm was proposed for a vehicular ad hoc network (VANET) with RSUs. Because user characteristics do not change easily, the social characteristics of vehicle users and traffic patterns were considered to identify vehicles with similar routes. In [14], a caching method was proposed based on content popularity in VANET. It predicted the hot-spot region to which the vehicles would go through the past trajectory and used the vehicles that seemed to stay in the hot-spot region for a longer period of time as caching nodes. One of the disadvantages of VANET is that it lacked personal privacy because the personal vehicle directly delivers the content. The use of named data networking (NDN) helps to protect the privacy because it uses the named content more than host identifiers. The application of vehicular networks to NDN is called vehicular named data networking (VNDN). In [15], a cooperative caching approach was proposed based on clustering vehicles with similar mobility in a VNDN. A mobility prediction model was created through relationships with surrounding vehicles and was used for clustering formation. In [16], a popularity-incentive-based caching scheme was proposed for the VNDN. The Stackelberg game was used to take account of the different characteristics of individual vehicles.

The environment in which vehicles move on roads is complex, and changes rapidly over time. Reinforcement learning was used to identify complicated environments and propose effective caching algorithms. In [17], a cooperative caching strategy was proposed to store the content of vehicles and RSUs through a Q-learning algorithm. It reduced the interference by clustering nearby vehicles and limiting itself to only one vehicle that provided content inside the clustering. In addition, a request prediction model using the LSTM was used. Algorithms for simultaneously optimizing caching and offloading computational tasks have also been proposed. In [18], a method using a deep Q-network (DQN) was proposed to optimize the caching and computing resources together. The speed of vehicles, file size, backhaul capacity, and cloud resources were considered to minimize the communication, storage, and computational costs. In [19,20,21], deep reinforcement learning was applied to determine the content that had to be cached and offload computation to RSUs. In [22,23], a DDPG-based caching method that determines the content to be cached in both RSUs and vehicles and the bandwidth to be allocated was proposed. In [22], the authors considered the requested content and deadlines, size of the remaining content to be delivered, and vehicle location, with the goal of reducing both the content update cost and bandwidth usage cost. Because there was a difference in the vehicle’s speed and cache update speed, two different timescales were used. In [23], the authors considered the surrounding vehicles of each vehicle, data rate of caching nodes, and directions of vehicle movement with the aim of minimizing the content transmission delay. To maintain the delay constraint, if delivery was not performed within the time limit, a penalty was imposed.

The above studies were conducted in environments where the RSU ranges did not overlap or the overlapping areas were not specifically considered. However, in an urban environment, the density of infrastructure is very high; thus, areas with overlapping service ranges must be created. If the redundancy of cached contents between overlapping RSUs is reduced and various contents are cached, vehicle users are more likely to access various contents. As the size of the content obtained from the caching nodes increases, the latency decreases, and the delay constraint is satisfied. Therefore, in this study, we propose a caching algorithm that stores content in vehicles and RSUs, by considering overlapping RSUs.

## 3. System Model

In this section, the overall framework of the caching method in vehicular networks is defined.

### 3.1. Network Model

We considered an urban environment. The network consists of one MBS and N RSUs, V vehicles. Because the coverage of the MBS is usually wider than these of the RSUs, we assumed that the MBS can cover the entire area of the environment, which means the MBS could always be connected from a vehicle in any position in the area. The MBS provides seamless service to vehicle users. We assumed that the MBS is located at the center of the network, and the RSUs are installed randomly. The MBS was wired for all RSUs. The RSUs were connected through a backhaul link. Vehicles communicated wirelessly with MBS and RSUs. MEC can provide the cache and the computation function; the OBU in the vehicle has the ability to communicate with RSUs and other vehicles and provides limited cacheable ability [24]. By installing MEC on the RSUs, both the RSUs and the vehicles have space to store content. Therefore, RSUs and vehicles were all used as caching nodes. If content requests are distributed to the caching nodes, the backhaul burden can be reduced. Even distribution of requests also helps the requested content delivered within the transmission deadline. M is the total number of caching nodes, M=N+V. The MBS acts as a content provider and has all the content. The MEC server of the RSU n, n∈N and OBU of vehicle v, v∈V have limited storage capacity, which is expressed as $C_{n}$ and $C_{v}$, respectively. The sum of the content sizes stored in each caching node cannot exceed its limited storage capacity.

**Figure 1:**
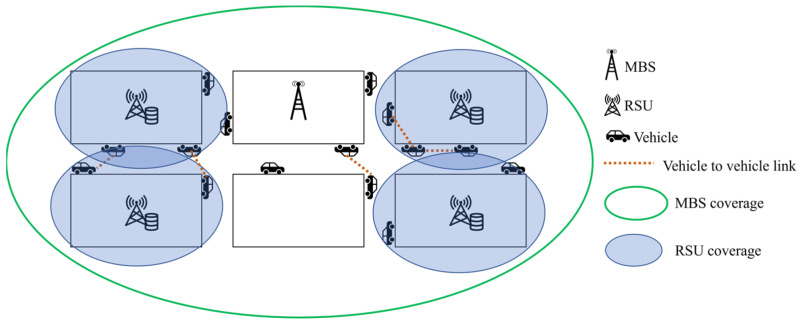
Caching framework in the vehicular network with different types of caching nodes.

Figure 1 describes the framework of content requests and content delivery in an urban environment. Some vehicles request content, and others serve as caching nodes to deliver the content to other vehicles in each time slot. We assumed that a vehicle requesting content cannot simultaneously provide the content to other vehicles. When a vehicle requests content, it checks the amount of content available through its OBU, and identifies the requirement of additional content. Additional content could be delivered through a caching node that has the strongest signal within a connectable distance. The content may be transmitted only from one of the caching nodes, vehicles, or RSUs. If the size received through the caching node is smaller than the total size of the requested content, then the remaining amount can be delivered through the MBS. If there are no caching nodes accessible from the vehicle, MBS could deliver the requested content to the vehicle.

### 3.2. Content Request Model

K contents are present in the MBS content library. Each content has a size of $z_{k}$ and delay constraint $e_{k}$, k∈K. Some vehicles request content during each time slot. Among V vehicles, vehicles with a ρ ratio request content and vehicles with a 1 − ρ ratio serve as caching nodes. The vehicles would either request or provide content; this was randomly determined during every time t. A vehicle can request only one content item at a time.

We assumed that content requests are generated through the zipf distribution. It is known that file requests in many web caching studies follow the zipf distribution [25]. In addition, the zipf distribution is also used in caching techniques for mobile users [26,27] and vehicles [28,29]. $\phi_{k}$ is the popularity of content k, and is expressed as follows [25]:

(1) $$\phi_{k}=\sum_{i=k}^{K}{\left(\frac{1}{i^{\alpha }}\right)}^{-1}\times \frac{1}{i^{\alpha }}$$

where α is more than zero and less than one. k is the popularity order of the content. The contents are sorted by popularity, so k is the content index. α affects the evenness of the content popularity. When α is smaller than 1, the popularity of the content is concentrated on less content items with an decrease in α. As α increases, the popularity of each piece of content becomes similar.

### 3.3. Caching Model

In this study, a coded caching technique is used. Coded caching uses network-coding techniques in which files are transmitted in an integrated and coded form. After receiving the coded packets, they are recovered to the original files [30]. Coded caching has the advantage of increasing network throughput and reducing delivery latency [30]. Maximum distance separable (MDS) codes were adapted to consider the high mobility of vehicles. Due to the high mobility of the vehicle, the connection time between the caching node and the vehicle requesting content is very short. It is difficult to deliver the entire content to the vehicle from the caching node before it leaves the service area of the caching node. Therefore, caching content in segments is more efficient in vehicular networks and MDS codes help keep the content fragmented. For simplicity, it is assumed that the receiver can recover the original content if more than the entire size of the content is delivered regardless of the order [31,32]. The total size of one content item in the caching nodes is set to one, and the content is cached in divided form into ten pieces. The ratio of content *k* cached in caching node m at time t, $x_{m,k}^{t}$, has one of the values {0, 0.1, 0.2, 0.3, 0.4, 0.5, 0.6, 0.7, 0.8, 0.9, 1}. The sum of the contents cached on each node does not exceed its storage capacity.

(2) $$\sum_{k=1}^{K}z_{k}x_{m,k}^{t}\;\leq \;C_{m}$$

(3) $$x_{m,k}^{t}\;\in \left\{0,0.1,0.2,0.3,0.4,0.5,0.6,0.7,0.8,0.9,1\right\}$$

### 3.4. Communication Model

Vehicles that require content can connect to only one caching node during every time slot. Vehicles in the service area of the RSU can be connected to the RSU. Vehicles within a communication distance can be connected to one another. If the content can be delivered from multiple caching nodes, the node with the highest data rate is selected. The RSUs check the information of the vehicle, such as the cached content and distance between vehicles, in its service area, and periodically send a status message to the MBS. The MBS recognizes that is happening in its service area. RSUs transmit content to multiple vehicles at time t. The RSU transmits the cached content to one vehicle and then sequentially delivers the requested content to other vehicles. MBS and all caching nodes transmit the content to vehicles by applying orthogonal frequency division multiple access (OFDMA) It is assumed that the link between the vehicle requiring the content and the caching node is continuously connected for a time t.

To receive the content requested by the vehicle from another vehicle, it must be within the communication range $D_{v}.\;d_{j,i}$ is the distance between vehicle i which requests content, and vehicle j  which provides the content. When $d_{j,i}\leq D_{v}$, the two vehicles communicate. The radius $D_{n}$ of the RSU n is a service area. If the distance between vehicle i and RSU n is smaller than $D_{n}$, vehicle i can receive the content from RSU n. It is assumed that a vehicle at any location can always connect to the MBS. The signal to noise ratio (SNR) between vehicles i and caching node m is calculated as follows:

(4) $${\mathrm{SNR}}_{m,i}=\frac{power_{m}g_{m,i}d_{m,i}{}^{-\tau }}{\sigma^{2}}.$$

$power_{m}$ is the transmission power of caching node m. $g_{m,i}$ is the channel gain between vehicle i and caching node m. $d_{m,i}$ is the distance between caching node m and request vehicle i. τ is the path-loss exponent. $\sigma^{2}$ the Gaussian noise. A data rate $dr_{m,i}$ for the caching node m to deliver content to vehicle i is as follows:

(5) $$dr_{m,i}=b_{m}\log_{2}\left(1+{\mathrm{SNR}}_{m,i}\right),$$

$b_{m}$ is the bandwidth of caching node m. When vehicle i requests content k and the ratio of content k cached by caching node m is $x_{m,k}$, the delay in content delivery from caching node m is as follows:

(6) $$delay_{i,m,k}=\frac{z_{k}x_{m,k}}{dr_{m,i}},$$

$z_{k}$ is the full size of content k and $z_{k}x_{n,k}^{t}$ is the real size content k cached in the caching node m at time t.

After receiving the content through the caching node, if an additional download is required, the MBS sends the remaining size of the requested content. The delay at which content k requested by vehicle i is received from the MBS is as follows:

(7) $$delay_{i,MBS,k}=\frac{z_{k}\left(1-w_{v,i}x_{v,i}-w_{n,i}x_{n,i}\right)}{dr_{MBS,i}}$$

$w_{v,i}$ is an indicator of content transmission between vehicle v and the vehicle i. If vehicle i receives the content from vehicle v, $w_{v,i}=1$ and otherwise, 0. Similarly, if vehicle i receives the content requested from RSU n, $w_{n,i}=1$ and otherwise 0. Because the content can be transmitted from only one caching node, $w_{v,i}+w_{n,i}=1$. $dr_{MBS,i}$ is the data rate of the MBS used for delivery content to the vehicle, i. It is calculated as in (5). The time taken to download the request by vehicle i is as follows:

(8) $$delay_{i}^{t}=w_{v,i}^{t}delay_{i,v}^{t}+w_{n,i}^{t}delay_{i,n}^{t}+delay_{i,MBS}^{t}.$$

### 3.5. Vehicular Mobility

Because the position of a vehicle changes over time, it is necessary to record the position for every time slot. The location of the vehicle is expressed in three positions. The first is the RSUs within the $D_{n}$ distance from the vehicle. Because there are overlapping RSUs, they can be expressed as several RSUs. The second is the future location of the vehicle, predicted using the LSTM model. This is expressed based on the RSU to which the vehicles belong. The third is the latitude and longitude coordinates of the current time period. They are required to measure the distance between vehicles, between the vehicle and RSU, and between the vehicle and MBS.

## 4. Deep Reinforcement Learning for Caching Strategy

A significant amount of information regarding the environment is required to determine the content to be stored in the caching nodes. For example, the bandwidth between vehicles and RSUs, the routes of vehicles and the popularity of requested contents are usually used as the information of the environment [9,11]. Using the LSTM model, the subsequent positions of the vehicles can be predicted. The number of vehicles in each RSU’s area was calculated using this prediction. In addition to the prediction, the popularity of content and links between caching nodes and requesters were considered to determine the content to be cached using DDPG.

### 4.1. Mobility Prediction of Vehicles with LSTM

The type of content requested varies depending on the number of requests. If there are a large number of requests, the highly popular content is mainly requested. However, if there are few requests, users want to receive the diverse contents that are relatively unpopular [8]. As the number of vehicles increases, the number of requests also increases proportionally. Therefore, if the number of vehicles staying in the service area for each RSU can be known in advance, it helps to determine the content that has to be cached.

Our proposed caching algorithm is not sensitive to the prediction accuracy. This is because the predicted vehicle location was used to know if there were possible connections in the common coverage of the RSUs or vehicles. We applied LSTM [33] as an example of a number of prediction models to predict vehicle locations, but models such as Markov Chain and Gaussian Mixture Model can also be used depending on the situation.

**Figure 2:**
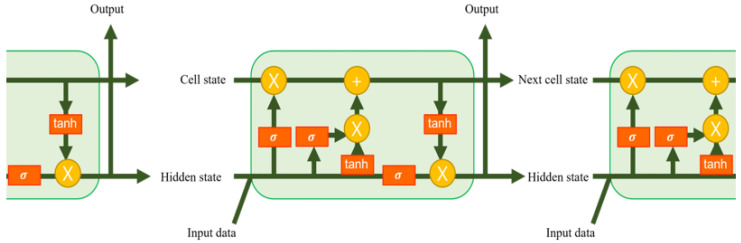
Architecture of LSTM.

LSTM predicted the location of vehicles, and the number of vehicles located in the coverage of RSUs was calculated based on the prediction. In other words, LSTM is used to anticipate how many requests each RSU would have. The LSTM has the following characteristics. First, LSTM usually predicts the future data on sequential types solely from vehicle position data. Additional information such as the personal data of users is not required for prediction. Second, LSTM learns historical information better than Recurrent Neural Networks (RNN). Figure 2 depicts the structure of the LSTM. The gates present inside the LSTM cell decide whether to transfer the input information to the subsequent cell or not, so that both long- and short-term memories can be obtained. As an input to the LSTM, latitude and longitude coordinates of vehicle v $\left\{\left[lat_{v},\;lon_{v}\right]\right\}$ are used. The output of LSTM is the subsequent time location of the vehicle. Once the subsequent position is predicted for all vehicles, the number of vehicles located in each RSU in the future can be calculated.

### 4.2. Deep Deterministic Policy Gradient

Reinforcement learning is a type of machine learning technique in which an agent learns to achieve a desirable goal. Reinforcement learning can be divided into value-based and policy-based methods. Policy-based reinforcement learning has the advantage that it is easier to learn probabilistic policies than value-based methods, and learning is possible in continuous environments. DDPG [34] is a policy-based method.

**Figure 3:**
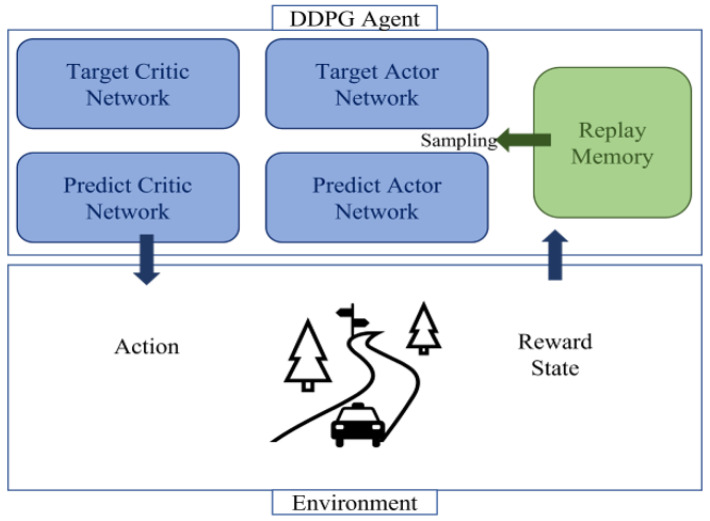
Architecture of DDPG.

DDPG uses two types of networks: a critic network and an actor network. Figure 3 illustrates the architecture of DDPG. The critic network serves to evaluate the action based on the value of the action selected by the agent. As the agent interacts with the environment, it stores the observed state s, selected action a, reward r obtained through the action, and new state s′ in the replay buffer D. The agent samples data in minibatches from the replay buffer to train the critic network. To update the critical network, it trains to reduce the difference from the output of the target neural network.

(9) $$L_{i}\left(\theta_{i}\right)=E_{\left(s,a,r,s\prime \right)\sim u\left(D\right)}\;\left[(r+\gamma Q{\left(s\prime ,\mu \left(s\prime ;\varphi^{-}\right);\theta^{-}-Q\left(s,a;\theta_{i}\right)\right)}^{2}\;\right]$$

$L_{i}\left(\theta_{i}\right)$ is the expected value of the difference between Q-value of the target critic network and Q-value of the train critic network [35]. (s,a,r,s′)~u(D) is the sampling through mini-batch data from the replay buffer  D. γ is the discount factor. $\theta_{i}$ is the train network parameter, and $\theta^{-}$ is the target critic network parameter, and these give the weights and bias. i is the parameter index. μ is the policy of the actor network and $\varphi^{-}$ is the parameter of the target actor network.

The actor network uses the state of the environment as the input and actions as the output to calculate the policy. The method of evaluating the policy of the actor network is to use the Q-value, which is the output of the critic network.

(10) $$J_{i}\left(\varphi_{i}\right)=E_{s\sim u\left(D\right)}\left[Q\left(s,\mu \left(s;\varphi \right);\theta \right)\right]$$

$J_{i}\left(\varphi_{i}\right)$ is the expected Q-value through an action selected according to the policy [34], and the actor network trains to make $J_{i}\left(\varphi_{i}\right)$ higher. s~u(D) denotes the states sampled from the replay buffer D. Policy learned through actor networks is deterministic, but this does not mean that the result is always correct. An appropriate exploration process is required to learn a suitable policy. In DDPG, exploration is performed using Gaussian noise. During the training process, Gaussian noise is added to the actions resulting from the actor network, further allowing the exploration of various actions.

The target networks of the critic and actor periodically update their parameters. This is because a new target value is required according to the training level while properly fixing the value. The soft update method is used to update the target network [34].

### 4.3. Caching Strategy with DDPG

There are various problems with transmitting content from remote servers. When the requests are focused on the remote server, the transmission speed decreases, further making it difficult to satisfy the delay constraint, and backhaul traffic increases. Caching content in RSUs and vehicles alleviates these problems. In this study, we propose a caching strategy to maximize the throughput of caching nodes consisting of RSUs and vehicles.

(11) $$P1:maximize\;throughput_{caching}$$

(12) $$subject\;to\;\sum_{m=1}^{M}z_{k}x_{m,k}^{t}\;\leq \;C_{m}$$

$throughpu_{caching}$ is the average content size delivered through the RSUs and vehicles, and not the MBS. There are M caching nodes, and $x_{m,k}^{t}$ is the size of content k cached in caching node m at time t expressed between zero and one. $z_{k}$ is the real size of content k. $C_{m}$ is the storage capacity of caching node m. The sum of the content stored in each caching node cannot exceed its storage capacity.

Because it is based on an urban environment, many RSUs are installed, and there are spaces where service areas overlap each other. The more varied the content in overlapping RSUs, the more content RSUs can provide to users. The system becomes very complicated because there are not only overlapping RSUs, but also moving vehicles. When a vehicle is not moving, it always accesses the same caching node. However, because the caching nodes that the moving vehicles can access and the bandwidth that the vehicles can use change over time in urban environments, the caching problem becomes complex. Compared to not overlapping RSUs, the caching problem becomes more difficult, addressing not only the contents that are cached in each RSU, but also the duplicated contents that are cached in multiple RSUs. DDPG was applied to such a time-varying dynamic environment because DDPG can effectively deal with high-dimensional problems. The agent of DDPG adapted the changing environments by expressing the observed changes as the state of MDP. The agent determines the types and sizes of content to store in each caching node to maximize the defined reward of MDP under the present state. It is assumed that an agent of the DDPG is on the MBS. The state of MDP can be expressed as follows:

(13) $$s^{t}=\left\{o_{y,k}^{t},p_{k}^{t},h_{n}^{t},l_{m}^{t}\right\}$$

$s^{t}$ is the state at time t. $o_{y,k}^{t}$ indicates the extent of overlapping RSUs cluster *y* cache content k at time t. RSUs with overlapping ranges are clustered to manage duplicated content in a space where the ranges of the RSUs overlap. There are Y clusters of duplicated RSUs. When the vehicle is in an area that can receive content from multiple RSUs, the hit ratio becomes high if the transmitted content is diversified. $o_{y,k}^{t}$ is calculated as follows:

(14) $$o_{y,k}^{t}=\sum_{i\in y}x_{k,i}^{t}$$

$p_{k}^{t}$ is the popularity of content k at time t and is calculated as follows:

(15) $$p_{k}^{t}=\sum_{k=1}^{K}q_{k}^{t}$$

$q_{k}^{t}$ is the number of contents k requested at time t. The content request was generated through zipf distribution in (1), and the agent identified the content requests as $p_{k}^{t}$. The higher the popularity, the higher is the probability of requesting the content, and it is advantageous to cache the content with high popularity. $h_{n}^{t}$ denotes the number of vehicles in RSU n at time t. This is because content with different popularity is requested depending on the number of requests from the vehicles. To calculate $h_{n}^{t}$, the LSTM output was used. Using vehicle data at time t−1, LSTM predicts each vehicle location at time t and $h_{n}^{t}$ can be computed. $h_{n}^{t}$ is computed as follows:

(16) $$h_{n}^{t}=\sum_{i=1}^{V}\mu_{i,n}^{t}$$

$\mu_{i,n}^{t}$ indicates whether vehicle i is in the service area of RSU n at time t. When $D_{n}$ is the service radius of RSU n, if the distance $d_{i,n}^{t}$ between the vehicle i and the RSU n is smaller than $D_{n}$, then $\mu_{i,n}^{t}$ = 1 and otherwise, 0. $l_{m}^{t}$ counts the number of connectable caching nodes m for each vehicle. When many demands are concentrated on one caching node, the amount of processing at one caching node increases, which makes it difficult to satisfy the delay constraint. $l_{m}^{t}$ is used to prevent the content requests from being concentrated in one caching node. $l_{m}^{t}$ is calculated as follows:

(17) $$l_{m}^{t}=\sum_{i=1}^{V}\eta_{\;i,m}^{t}$$

$\eta_{i,m}^{t}$ indicates that the vehicle i is within the communication range $D_{m}$ of the caching node m. If the distance $d_{i,m}^{t}$ between the vehicle i and the caching node m is smaller than $D_{m}$, $\eta_{i,m}^{t}=1$ and otherwise, 0. Both $h_{n}^{t}$ and $l_{m}^{t}$ use vehicle location information, with the difference that $h_{n}^{t}$ adopts future vehicle location and $l_{\;m}^{t}$ adopts current vehicle location. All states were used with normalization.

(18) $$a^{t}=\left\{x_{m,k}^{t}\right\}$$

$a^{t}$ refers to the action selected by the agent at time t. $x_{m,k}^{t}$ denotes the size of content k cached at caching node m at time t. It is expressed between zero and one because one piece of content can be cached by dividing it into ten pieces with MDS codes.

(19) $$r^{t}=\beta \times \frac{throughput_{caching}}{throughput_{MBS}}+\left(1-\beta \right)\times hit\;ratio$$

$r^{t}$ means the reward received by the action at time t. The reward is composed of two factors. One is the caching node throughput to the MBS throughput ratio and the other is the hit ratio. If the requested content is in the caching node, the hit is one; otherwise, it is zero. The hit ratio was calculated by dividing the sum of all hits by the total number of requests. Both factors were used to increase the throughput of the caching nodes while simultaneously guaranteeing the hit ratio. β means a weighting factor between zero and one. The reward was used for normalization.

## 5. Performance Evaluation

For the experimental analysis of the proposed algorithm, real vehicle data, Dataset of mobility traces in San Francisco, USA [36], was used. The location of the vehicle was recorded in latitude and longitude coordinates along with a timestamp in the dataset. The timestamps were arranged at regular intervals, and the distance traveled was adjusted according to the time intervals. The experimental space was assumed to be 2.5 km in width and 2.5 km in length and 36 RSUs were installed in the space. The experiments were conducted for 9 min by considering that the average vehicle speed was approximately 16 km/h. We configured that approximately 60% of the moving vehicles requested content, and remaining 40% of them acted as caching nodes. This is because in [22], the system cost was lowest when the requiring vehicle and the caching vehicle were in a ratio of 30 to 20. Whether to request content or serve as a caching node is randomly determined for each vehicle in each time slot. The bandwidth of the caching nodes and MBS was allocated to the connected requestor vehicles according to the situation. 500 episodes were used to train the DDPG agent as in [20]. The learning rate and the discount factor were configured as 0.0003 and 0.99, respectively. The parameters used in the experiments are listed in Table 1.

The algorithms of *Random*, cooperative content caching (*CCC*) [22] and edge caching with content delivery (*ECCD*) [23] were adopted for the performance comparison. The *Random* method randomly selects the type and size of content to be cached for each caching node. Both the *CCC* and *ECCD* algorithms adopted DDPG to solve the problem of content caching and bandwidth allocation of edge nodes. The goal of *CCC* is to minimize the storage cost consisting of the update cost and bandwidth usage of caching nodes. The update cost used in *CCC* refers to the difference of the number of contents that cached in caching node m between time t and time t+1. *CCC* also considers the failure to deliver within the delay constraint to minimize storage costs. In the original *CCC*, vehicles requesting content and vehicles providing content are completely separated; however, to compare performance in the same environment for our experiments, a certain percentage of vehicles are configured to request content during every time slot. The *CCC* considers bandwidth allocation of all caching nodes, but to make the system model similar to the one proposed in this method, the vehicle allowed only a one-to-one connection for the content requestor and content provider. The *ECCD* aims to minimize the overall content delivery time and uses the requested content popularity multiplied by the content delivery time as a penalty. Both *CCC* and *ECCD* stored the content as a whole, but MDS codes were applied for comparing it with the proposed algorithm. Regarding the bandwidth allocation of RSUs, the same method in the proposed algorithm was adopted in *CCC* and *ECCD*.

To compare the proposed caching strategy with *Random*, *CCC* and *ECCD*, three scenarios were designed. The first scenario is for the experiments to evaluate the performance on the number of vehicles. Because the size of the experimental space is fixed, as the number of vehicles in the space increases, the density of vehicles becomes high. When the number of vehicles increases, the number of caching nodes that vehicles can access is also increased. However, if a large number of vehicles generate requests simultaneously, it brings about a negative effect on the delay constraint satisfaction. Hence, this scenario is intended to show how many vehicles the caching methods could stand. The second scenario is for the experiments on the number of RSUs. As the number of RSUs decreases, the number of caching nodes that vehicles can connect with is decreased. The experiments in the second scenario are to find out the relationship between the density of RSUs and the performance. The third scenario is for the experiments on the capacity of RSUs. The performance of caching usually is affected by the storage capacity, and the experiments with third scenario seek to evaluate the capacity tolerance of caching methods.

The performance criteria are the throughput of caching nodes, delivery constraint satisfaction, and update cost. The throughput of the caching nodes indicates the average size of the content delivered from the caching nodes to each requesting vehicle in a time unit. The high throughput can be achieved as the requests are distributed to caching nodes, the burden of backhaul decreases and the transmission time is reduced. The throughput of caching nodes is calculated as follows:

(20) $$Throughput_{caching}=\frac{1}{F}\sum_{f=1}^{F}w_{m,f}^{t}x_{m,k}^{t}$$

$w_{m,f}^{t}$ indicates whether or not the requesting vehicle f is receiving from the caching node m at time t. If the vehicle f is receiving from the caching node m, $w_{m,f}^{t}$ = 1 and otherwise, 0. $x_{m,k}^{t}$ is the content k size that is cached in caching node m at time t and F is the number of requesting vehicles. By identifying the amount of content delivered by all caching nodes for each time slot, the throughput of caching nodes was measured. Because the goal of the proposed algorithm is to maximize the amount of content delivered from the caching nodes, we can verify if the goal has been achieved. Delay constraint satisfaction means the ratio at which the entire content size is delivered within the time limit for which content k had to be delivered. The degree of delay constraint satisfaction is calculated as follows:

(21) $$Delay\;constraint\;satisfaction=\frac{1}{F}\sum_{f=1}^{F}\varpi_{f}^{t},$$

(22) $$\varpi_{f}^{t}=\{\begin{array}{l} 1,\;if\;delay_{f}^{t}\leq deadline\\ 0\;\;\;\;\;\;\;\;\;\;\;\;\;\;\;\;\;\;\;\;,\;otherwise \end{array}$$

$\varpi_{f}^{t}$ indicates whether the vehicle f is received the requested content within the delay constraint. The $delay_{f}^{t}$ is the time taken for the vehicle f to receive the requested content at time t and can be calculated as (8). The delay constraint, deadline indicates the maximum delay time that the end-to-end delay cannot exceed when content was received. If content requests are properly distributed to caching nodes rather than being concentrated on the MBS, the delivery latency is reduced, and the delay constraint satisfaction increases. A high delay constraint satisfaction has a positive effect on Quality of Service (QoS) of the requesters. The update cost refers to the average of the cached content sizes that are additionally stored or deleted when it becomes time *t* + 1 from time *t*. Updating the cached content significantly to match the time-varying environment helps to increase the hit ratio, but it places a burden on the backhaul links as the RSUs have to fetch additional content from the MBS. We tried to minimize the update cost while increasing the throughput of caching nodes. The update cost is calculated as follows:

(23) $$Update\;cost=\frac{1}{C_{m}}\sum_{m=1}^{M}\sum_{k=1}^{K}\left|x_{m,k}^{t+1}-x_{m,k}^{t}\right|\;$$

The vehicles and RSUs figured out their own updated contents and they were used to average the update cost for all caching nodes. The update cost in the proposed algorithm is different from that of *CCC* which only computes the number of changed contents.

**Figure 4:**
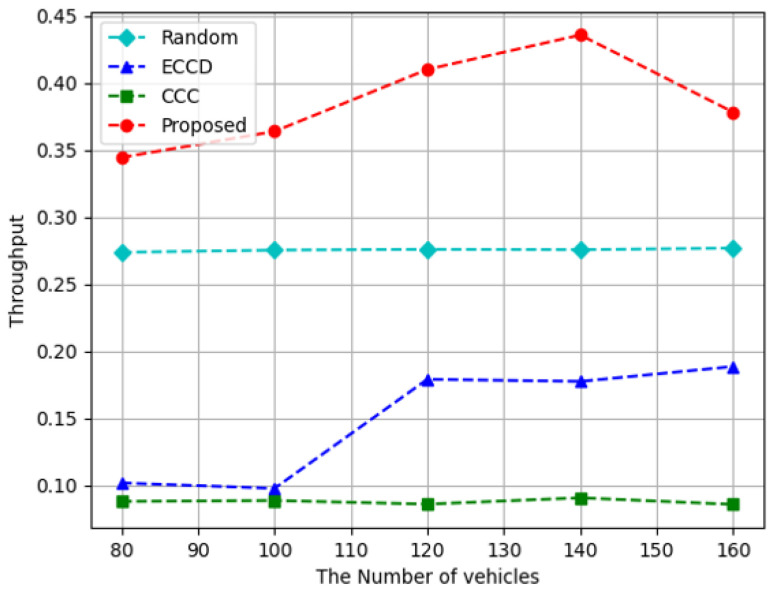
Throughput according to the increase in the number of vehicles.

**Figure 5:**
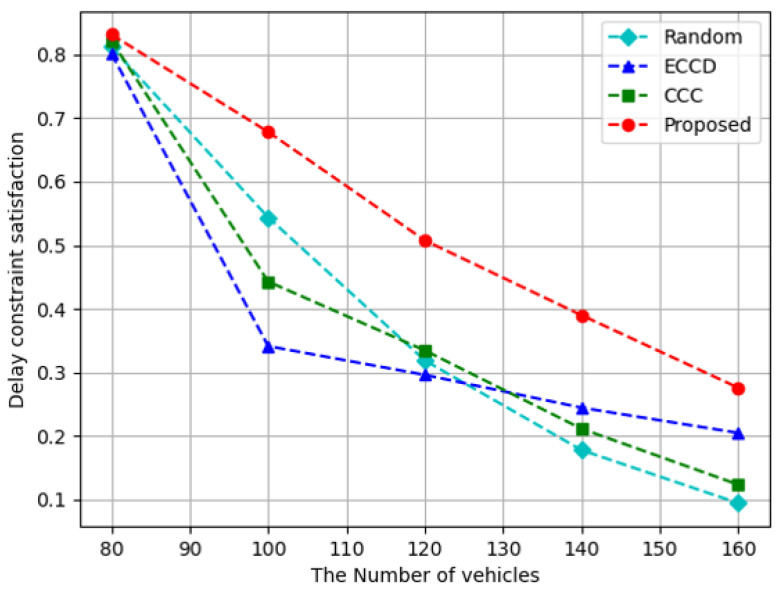
Delay constraint satisfaction with respect to the increase in the number of vehicles.

**Figure 6:**
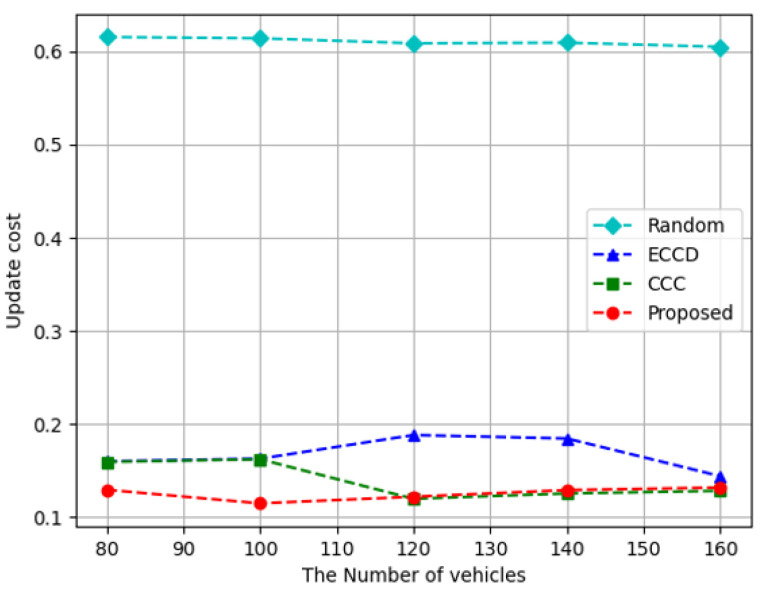
Update cost according to the increase in the number of vehicles.

Figure 4, Figure 5 and Figure 6 depict the results of the measurement while changing the number of vehicles used in the experimental environment. As the number of vehicles increases, the requests for content increase, placing a load on the system. On the other hand, when the number of vehicles increases, the number of caching nodes increases, further increasing the space for caching content. Figure 4 depicts the result of measuring the caching node throughput while increasing the number of vehicles in the experimental environment. Compared to other algorithms, the proposed algorithm demonstrated the highest result because the agent of the proposed algorithm trains by considering the size of the content downloaded through caching nodes as a reward. In the proposed algorithm, when the number of vehicles is 160, the edge throughput decreased because more content was requested compared to what the system could handle. In *ECCD*, the throughput increases when there are 120 vehicles, and when there are more than 120 vehicles, it has a constant value. This means that when there are fewer than 120 vehicles, more content is delivered from the RSUs; however, when there are more than 120 vehicles, the amount of content delivered from the vehicle is larger than that of the RSU, resulting in an increase in throughput. At the same time, the number of vehicles requesting content also increased and there is no further increase in the total throughput. The *Random* and *CCC* methods were not significantly affected by the number of vehicles used. The reason is that *Random* does not consider the current situation. In the case of *CCC*, there are already enough vehicles in the environment; therefore, the number of vehicles does not affect the results. On average, the proposed algorithm performs approximately 1.4 times higher than *Random*, and approximately 2.7 and 4.3 times higher than *ECCD* and *CCC*, respectively.

Figure 5 depicts the results of measuring delay constraint satisfaction. The delay constraint satisfaction tended to decrease as the number of vehicles increased. This is because although the number of vehicles used as caching nodes increased, the latency increased because of the content requests that were concentrated in specific RSUs, because the total number of requests increased more sharply. Comparison algorithms often cache a few contents in an excessively larger size or a lot of content in a significantly smaller size, which results in a long latency. In contrast, the proposed algorithm caches content to an appropriate size and distributes content requests. The proposed algorithm performs on an average 1.7 times higher than *Random*, and 1.5 and 1.6 times higher than *ECCD* and *CCC*, respectively.

Figure 6 depicts the results of measuring the update cost according to the number of vehicles. In the case of the proposed algorithm and *Random* method, consistent results were obtained regardless of the number of vehicles. This implies that the update cost of each caching node is not affected by the number of vehicles, and the update cost of caching the RSUs and vehicles is constant. The proposed algorithm had the lowest cost, and the reason is that the proposed algorithm has RSUs and vehicles that rarely varied the cached content. However, *ECCD* tends to decrease when there are 160 vehicles and *CCC* tends to decrease when there are 120 vehicles or more, which means that the update cost in vehicles decreases when the number of vehicles increases. In *ECCD* and *CCC*, as the number of vehicles increases various contents could be accessed without updating the stored contents in vehicles because a large number of vehicles were used as caching nodes around the requestor. The proposed algorithm demonstrated an average reduction of approximately 79% with *Random*, about 24% and 8% with *ECCD* and *CCC*, in terms of update cost.

**Figure 7:**
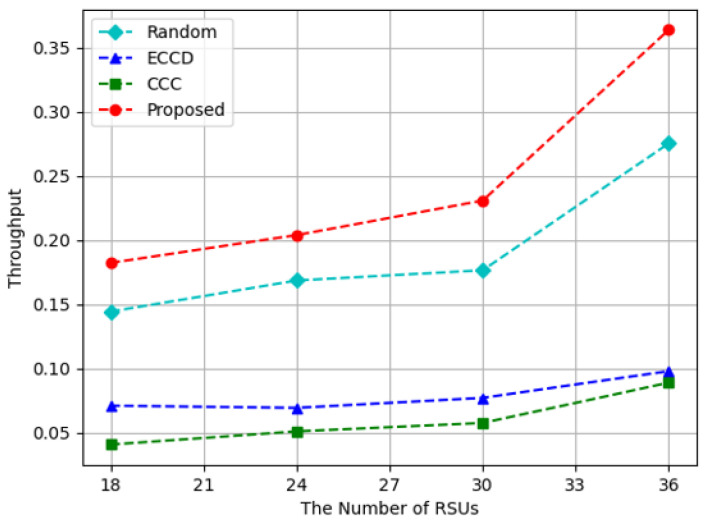
Throughput with respect to the increase in the number of RSUs.

**Figure 8:**
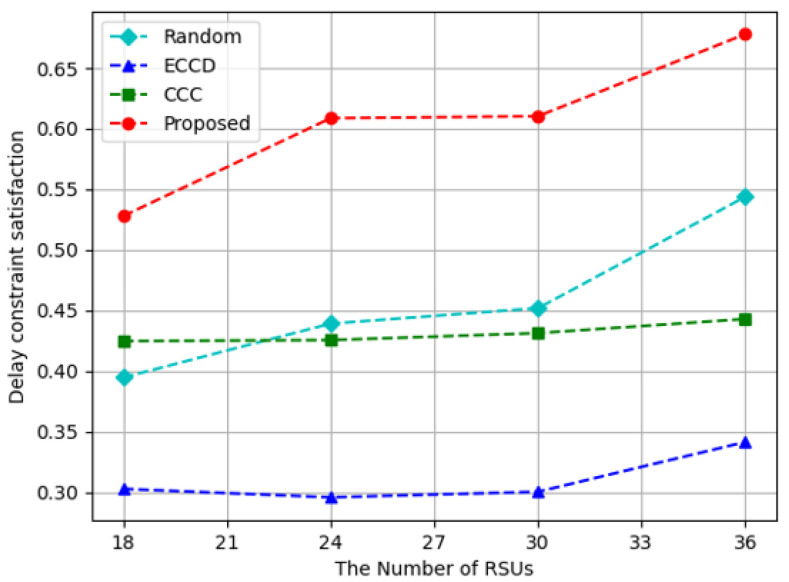
Delay constraint satisfaction with respect to the increase in the number of RSUs.

**Figure 9:**
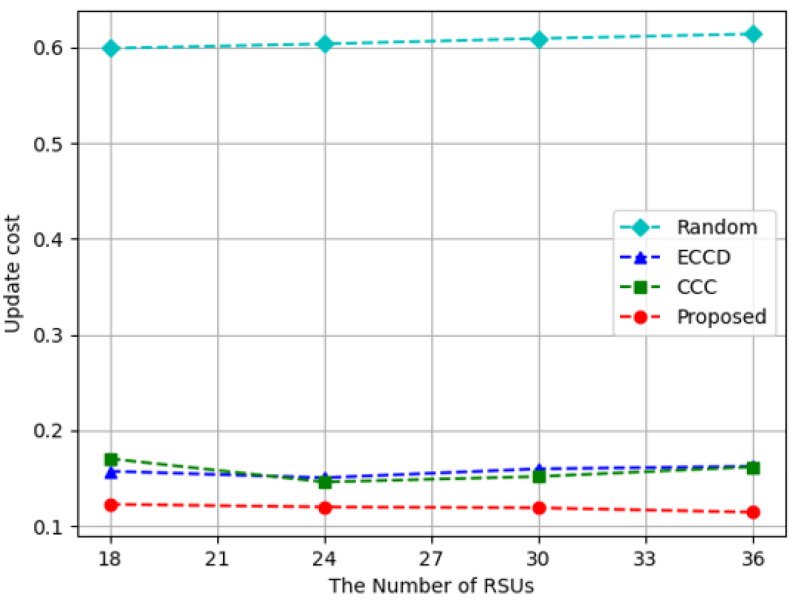
Update cost with respect to the increase in the number of RSUs.

Figure 7, Figure 8 and Figure 9 show the experimental results while increasing the number of RSUs from 18 to 36. Because the size of the experimental space was fixed, RSUs become sparser in the experimental space when the number of RSUs is decreased. If the number of RSUs in the space is increased, it means that RSUs are installed densely. In the space with 36 RSUs, vehicles can connect with at least one RSU wherever the vehicles are located. When the number of RSUs is 18, 24, or 30, there may not be an RSU that could be connected with the vehicles that requested content. Figure 7 shows the throughput of caching nodes according to the number of RSUs. The proposed algorithm and *Random* method had the explosive results when the number of RSUs was 36. When there were 36 RSUs in the experiment, the throughput was only affected by whether the requested content was cached or not. However, when the number of RSUs was less than 36, there were some cases where there was no RSU accessible from the requesting vehicles, resulting in low throughput. *ECCD* was not significantly affected by the number of RSUs because the amount of content transmitted from RSUs was smaller than the amount of content transmitted from other vehicles. In terms of the throughput, the proposed algorithm showed about 27%, 200%, and 314% better results than *Random*, *ECCD*, and *CCC*.

Figure 8 shows the results of measuring the delay constraint satisfaction according to the number of RSUs. The proposed algorithm showed similar results when 24 and 30 RSUs were installed because the size of area covered by all RSUs in the experimental space was similar. When there were 18 RSUs to be installed, the size of area that could be connected with RSUs is decreased, so content requests were concentrated on a small number of caching nodes, reducing the delay constraint satisfaction. When there were 36 RSUs, RSUs could be accessed from all experimental space, and content requests were distributed to several caching nodes, showing high delay constraint satisfaction. The *ECCD* and *Random* methods had the increasing delay constraint satisfaction as the number of RSUs was raised. In the case of *CCC*, the delay constraint satisfaction increased a little according to the number of RSUs. *CCC* was less affected by the number of RSUs because the amount of content delivered from vehicles is larger than from RSUs. In terms of delay constraint satisfaction, the proposed algorithm performed on average 33%, 95%, and 40% higher than *Random*, *ECCD*, and *CCC*, respectively.

Figure 9 depicts the results of measuring the update cost according to the number of RSUs. For all caching methods, the update cost is constant regardless of the number of RSUs, meaning that the number of RSUs does not affect the degree of the variation of cached content in each caching node. The *Random* method had a relatively high update cost compared to other caching algorithms. This is because other caching techniques except for *Random* all used reinforcement learning to cache content appropriately according to the environment, while *Random* method determined the type and size of content to be cached randomly regardless of the current situation of the environment. The proposed algorithm showed a lower update cost than *ECCD* and *CCC* and the reason is that the proposed algorithm had little change in the cached content over time. In the proposed algorithm, LSTM predicted future locations of vehicles and it was known in advance how many vehicles would be located in the coverages of RSUs. This information helped the proposed algorithm to consider the future situation when deciding which content to cache, so that the cached content did not have to change much when faced with the future. The proposed algorithm decreased the update cost on average by 80% compared to *Random*, 24% compared to *ECCD*, and 24% compared to *CCC*.

**Figure 10:**
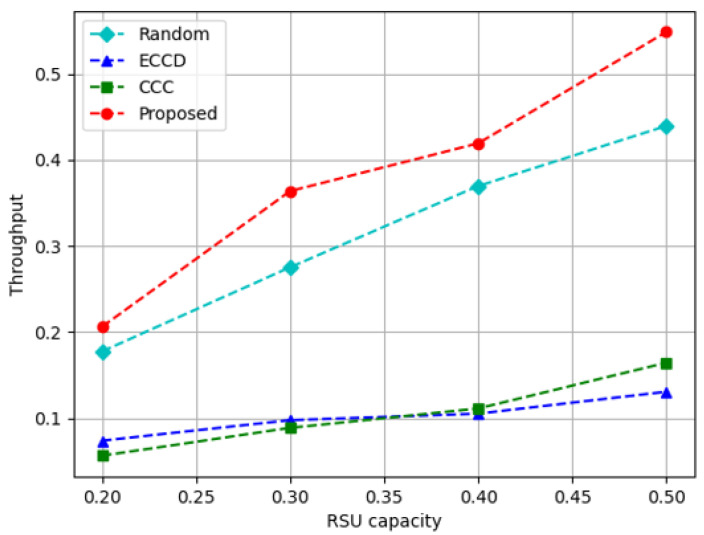
Edge throughput with respect to the increase in the capacity of RSUs.

**Figure 11:**
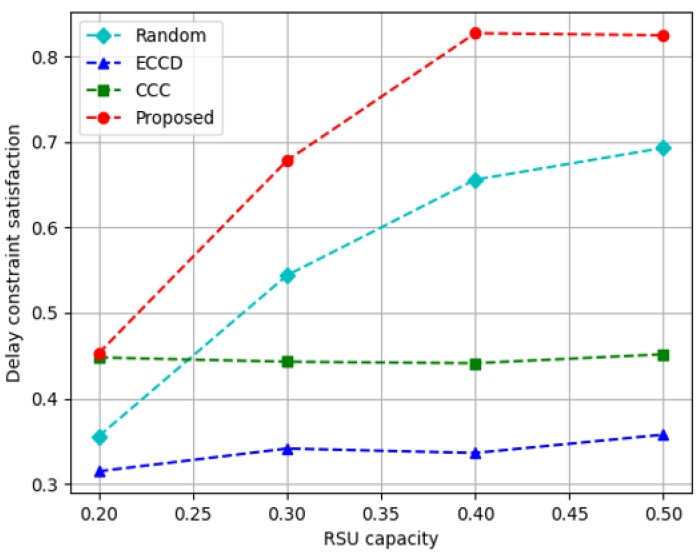
Delay constraint satisfaction according to the increase in the capacity of RSUs.

**Figure 12:**
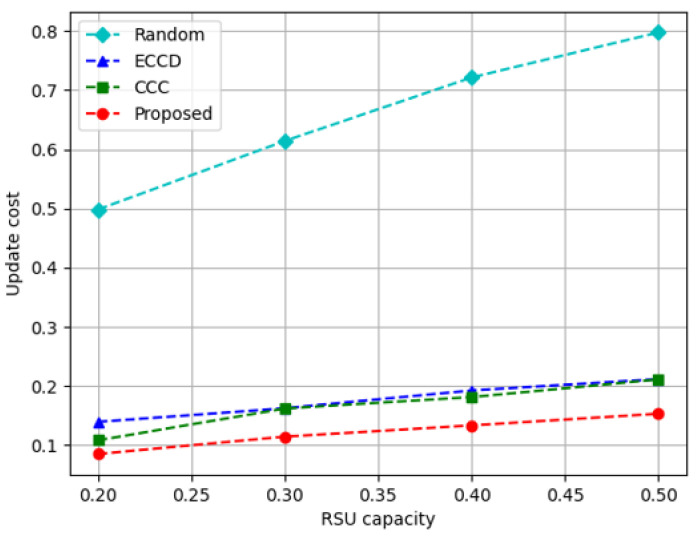
Update cost according to the increase in the capacity of RSUs.

Figure 10, Figure 11 and Figure 12 depict the performance measured while increasing the storage capacity of the RSU. If the capacity of the RSU is large, there is a large amount of content that can be cached, but there is a limit to the size that can be transmitted within the constraint time. The capacity of the RSU is expressed as the ratio of the size that can be stored in the RSU out of the total size of the content in the content library. Figure 10 depicts the results of measuring the caching node throughput according to the increase in the storage capacity of the RSU. In the proposed and comparison methods, as the capacity of the RSU increases, the content stored in the RSU increases, and thus, the caching node throughput increases. Caching a small amount of content to larger size results in lower performance, such as *ECCD* and *CCC*. Relatively, the proposed algorithm and *Random* cache a large amount of content with a small size, which helps the requestors to obtain content from caching nodes. On average, the proposed algorithm increased performance approximately 1.2 times more than *Random*, approximately 3.6 and 3.7 times more than *ECCD* and *CCC*, respectively.

Figure 11 depicts the results of the delay constraint satisfaction according to the RSU storage capacity. The proposed algorithm and *Random* method increased satisfaction significantly as the storage capacity of RSUs increased, whereas *ECCD* and *CCC* increased less. The agents of *ECCD* and *CCC* selected the caching nodes that transmit the requested content based on their own rewards. However, the proposed method always selected a caching node that provided content according to the size of the data rate between the caching node and the content requestor. A high data rate has a positive effect on delay constraint satisfaction. The proposed method demonstrated that the delay constraint satisfaction is increased on average by approximately 24% compared to *Random*, approximately 100% compared to *ECCD*, and approximately 56% compared to *CCC*.

In Figure 12, the result of calculating the update cost according to the RSU storage capacity is depicted. Compared to the methods using DDPG, *Random* has a higher value because the agent of DDPG observes the environment to determine caching, whereas *Random* decides the content to be cached regardless of the environment. The proposed method has the lowest cost because it does not frequently modify the cached content in RSUs. *CCC* has the second-smallest value because it regards the update cost as one part of the DDPG reward. The proposed algorithm decreases update cost by about 81% compared to *Random*, approximately 31% compared to *ECCD*, and approximately 26% compared to *CCC* in terms of update cost.

Through several experiments in different scenarios, the proposed algorithm showed higher performance in terms of the throughput, the delay constraint satisfaction, and the update cost. In terms of the throughput, the proposed method had better performance because it predicted the number of vehicles staying in the service area for the RSUs and computed the size of cached content with consideration of the overlapped service areas of RSUs unlike other algorithms. High throughput led to high delay constraint satisfaction. In the case of the update cost, the proposed caching strategy had a smaller size that changed over time as compared to the other algorithms. This was because the agent of the proposed algorithm could recognize the future situation through LSTM in the current situation.

## 6. Conclusions

Recently, as the amount of service content that needs to be delivered from the remote server to the vehicles has increased, the processing burden of the remote server and the transmission latency from the remote server to each vehicle have increased accordingly. Therefore, in this study, we proposed an algorithm that maximizes the throughput of caching nodes by using RSUs and vehicles as caching nodes because the distribution of content requests to caching nodes can reduce the content delivery time and backhaul burdens. This method allocated the content requests to caching nodes rather than concentrating on MBS, so the burden on backhaul became low and the transmission latency became short. We used the LSTM to predict the future location of the vehicle, which helped to determine the number of vehicles in each RSU. The output of LSTM is the future location of vehicles and with this information the number of vehicles was predicted. DDPG was applied to handle this complicated environment to decide where and how much content to cache. Experiments in various scenarios demonstrate that the proposed algorithm performs better in terms of the caching node throughput, delay constraint satisfaction, and update cost. The experimental results with varying the number of vehicles demonstrated that the proposed algorithm had about 2.8 times higher throughput, about 1.6 times higher delay constraint satisfaction, and about 0.37 times lower update cost than the compared algorithms on average. In the experiments on the RSU density, the proposed strategy had about 2.8 times higher throughput, about 1.5 times higher delay constraint satisfaction, and about 0.42 times lower update cost than these of the other algorithms on average. In the experiments on the caching storage tolerance, the results showed that the proposed algorithm increased the throughput approximately 2.8 times and the delay constraint satisfaction about 1.6 times, and had about 0.46 times lower update cost compared to the other algorithms on average.

In future work, it will be possible to expand the study to optimize caching with energy efficiency. Because vehicles have limited energy supply, it is important to enhance the energy efficiency. Moreover, if the energy consumption of RSUs decreases, the maintenance costs can be reduced.

## Figures and Tables

**Table 1:** Experiment parameters.

Parameter	Value
Number of RSUs	[18, 36]
Number of vehicles	[80, 160]
Number of contents	10
Bandwidth of MBS	20 MHz
Bandwidth of RSU	10 MHz
Bandwidth of vehicle	5 MHz
Zipf parameter (α)	0.56
Weighting factor (β)	0.8

## Data Availability

Michal Piorkowski, Natasa Sarafijanovic-Djukic, Matthias Grossglauser, February 2009, CRAWDAD dataset epfl/mobility (v. 2009-02-24), downloaded from https://crawdad.org/epfl/mobility/20090224, https://doi.org/10.15783/C7J010, accessed on 23 September 2022.
